# Patient and Context Factors in the Adoption of Active Surveillance for Low-Risk Prostate Cancer

**DOI:** 10.1001/jamanetworkopen.2023.38039

**Published:** 2023-10-17

**Authors:** Giovannino Ciccone, Stefano De Luca, Marco Oderda, Fernando Munoz, Marco Krengli, Simona Allis, Carlo Giuliano Baima, Maurizio Barale, Sara Bartoncini, Debora Beldì, Luca Bellei, Andrea Rocco Bellissimo, Diego Bernardi, Giorgio Biamino, Michele Billia, Roberto Borsa, Domenico Cante, Emanuele Castelli, Giovanni Cattaneo, Danilo Centrella, Devis Collura, Pietro Coppola, Ettore Dalmasso, Andrea Di Stasio, Giuseppe Fasolis, Michele Fiorio, Elisabetta Garibaldi, Giuseppe Girelli, Daniele Griffa, Stefano Guercio, Roberto Migliari, Luca Molinaro, Franco Montefiore, Gabriele Montefusco, Maurizio Moroni, Giovanni Muto, Francesca Ponti di Sant’Angelo, Luca Ruggiero, Maria Grazia Ruo Redda, Armando Serao, Maria Sara Squeo, Salvatore Stancati, Domenico Surleti, Francesco Varvello, Alessandro Volpe, Stefano Zaramella, Giovanni Zarrelli, Andrea Zitella, Enrico Bollito, Paolo Gontero, Francesco Porpiglia, Claudia Galassi, Oscar Bertetto

**Affiliations:** 1 Epidemiologia Clinica e Valutativa, AOU Città della Salute e della Scienza di Torino e CPO Piemonte, Torino, Italy; 2 Urologia, AOU San Luigi Gonzaga e Università di Torino, Orbassano, Italy; 3 Urologia, AOU Città della Salute e della Scienza e Università di Torino, Torino, Italy; 4 Radioterapia, PO Umberto Parini, Aosta, Italy; 5 Radioterapia, AOU Maggiore della Carità e Università del Piemonte Orientale, Novara, Italy; 6 Radioterapia, AOU San Luigi Gonzaga, Orbassano, Italy; 7 Urologia, Ospedali Riuniti ASL TO4, Ciriè, Italy; 8 Urologia, AO Ordine Mauriziano, Torino, Italy; 9 Radioterapia, AOU Città della Salute e della Scienza e Università di Torino, Torino, Italy; 10 Urologia, Ospedali Riuniti ASL TO4, Ivrea, Italy; 11 Rete Oncologica del Piemonte e Valle d’Aosta, AOU Città della Salute e della Scienza di Torino, Torino, Italy; 12 Urologia, AO Santa Croce e Carle, Cuneo, Italy; 13 Urologia, PO Cardinal Massaia, Asti, Italy; 14 Urologia, AOU Maggiore della Carità e Università del Piemonte Orientale, Novara, Italy; 15 Urologia, PO SS Annunziata, Savigliano, Italy; 16 Radioterapia, ASL TO4, Ospedale di Ivrea, Ivrea, Italy; 17 Urologia, PO Umberto Parini, Aosta, Italy; 18 Urologia, PO San Biagio, Domodossola, Italy; 19 Urologia, PO Humanitas Gradenigo, Torino, Italy; 20 Urologia, AO SS Antonio e Biagio e Cesare Arrigo, Alessandria, Italy; 21 Urologia, PO Michele e Pietro Ferrero, Verduno, Italy; 22 Urologia, PO San Giovanni Bosco, Torino, Italy; 23 Radioterapia, Istituto di Candiolo-Fondazione del Piemonte per l’Oncologia (FPO), IRCCS, Candiolo, Italy; 24 Radioterapia, PO Nuovo Ospedale degli Infermi, Ponderano, Italy; 25 Urologia, PO Edoardo Agnelli, Penerolo, Italy; 26 Anatomia Patologica 1U, AOU Città della Salute e della Scienza di Torino, Torino, Italy; 27 Urologia, PO San Giacomo, Novi Ligure, Italy; 28 Urologia, PO Maria Vittoria, Torino, Italy; 29 Urologia, Ospedale Maria Pia, Torino, Italy; 30 Radioterapia, AO Ordine Mauriziano e Università di Torino, Torino, Italy; 31 Urologia, PO Martini, Torino, Italy; 32 Urologia, PO Rivoli, Rivoli, Italy; 33 Urologia, PO Nuovo Ospedale degli Infermi, Ponderano, Italy; 34 Anatomia Patologica, AOU San Luigi Gonzaga e Università di Torino, Orbassano, Italy

## Abstract

**Importance:**

Although active surveillance for patients with low-risk prostate cancer (LRPC) has been recommended for years, its adoption at the population level is often limited.

**Objective:**

To make active surveillance available for patients with LRPC using a research framework and to compare patient characteristics and clinical outcomes between those who receive active surveillance vs radical treatments at diagnosis.

**Design, Setting, and Participants:**

This population-based, prospective cohort study was designed by a large multidisciplinary group of specialists and patients’ representatives. The study was conducted within all 18 urology centers and 7 radiation oncology centers in the Piemonte and Valle d’Aosta Regional Oncology Network in Northwest Italy (approximate population, 4.5 million). Participants included patients with a new diagnosis of LRPC from June 2015 to December 2021. Data were analyzed from January to May 2023.

**Exposure:**

At diagnosis, all patients were informed of the available treatment options by the urologist and received an information leaflet describing the benefits and risks of active surveillance compared with active treatments, either radical prostatectomy (RP) or radiation treatment (RT). Patients choosing active surveillance were actively monitored with regular prostate-specific antigen testing, clinical examinations, and a rebiopsy at 12 months.

**Main Outcomes and Measures:**

Outcomes of interest were proportion of patients choosing active surveillance or radical treatments, overall survival, and, for patients in active surveillance, treatment-free survival. Comparisons were analyzed with multivariable logistic or Cox models, considering centers as clusters.

**Results:**

A total of 852 male patients (median [IQR] age, 70 [64-74] years) were included, and 706 patients (82.9%) chose active surveillance, with an increasing trend over time; 109 patients (12.8%) chose RP, and 37 patients (4.3%) chose RT. Median (IQR) follow-up was 57 (41-76) months. Worse prostate cancer prognostic factors were negatively associated with choosing active surveillance (eg, stage T2a vs T1c: odds ratio [OR], 0.51; 95% CI, 0.28-0.93), while patients who were older (eg, age ≥75 vs <65 years: OR, 4.27; 95% CI, 1.98-9.22), had higher comorbidity (Charlson Comorbidity Index ≥2 vs 0: OR, 1.98; 95% CI, 1.02-3.85), underwent an independent revision of the first prostate biopsy (OR, 2.35; 95% CI, 1.26-4.38) or underwent a multidisciplinary assessment (OR, 2.65; 95% CI, 1.38-5.11) were more likely to choose active surveillance vs active treatment. After adjustment, center at which a patient was treated continued to be an important factor in the choice of treatment (intraclass correlation coefficient, 18.6%). No differences were detected in overall survival between active treatment and active surveillance. Treatment-free survival in the active surveillance cohort was 59.0% (95% CI, 54.8%-62.9%) at 24 months, 54.5% (95% CI, 50.2%-58.6%) at 36 months, and 47.0% (95% CI, 42.2%-51.7%) at 48 months.

**Conclusions and Relevance:**

In this population-based cohort study of patients with LRPC, a research framework at system level as well as favorable prognostic factors, a multidisciplinary approach, and an independent review of the first prostate biopsy at patient-level were positively associated with high uptake of active surveillance, a practice largely underused before this study.

## Key Points

**Question:**

What characteristics among physicians and patients are associated with choosing active surveillance for low-risk prostate cancer within a research framework?

**Findings:**

In this cohort study that included 852 patients with low-risk prostate cancer, the proportion of patients choosing active surveillance was 82% and increased over time. A multidisciplinary discussion and a review of the diagnostic biopsy were associated with this choice.

**Meaning:**

These findings suggest that this research framework was associated with a large diffusion of active surveillance in clinical practice and high acceptance among patients.

## Teaser

This cohort study compares characteristics and clinical outcomes between patients with low-risk prostate cancer who receive active surveillance vs radical treatments.

## Introduction

The incidence of low-risk prostate cancer (LRPC) has increased over the past decades due to the widespread use of prostate-specific antigen (PSA) screening. To reduce overtreatment of indolent PC, an active surveillance strategy is strongly recommended as an appropriate management.^1,2,3,4,5,6,7^ The main purpose of active surveillance is to reduce the risk of treatment-related complications for patients with cancers that are not likely to progress, by delaying or avoiding definitive treatments in absence of signs of progression during a standardized follow-up.

Active surveillance has reassuring long-term results, derived from several cohort studies,^8^ and confirmed by randomized trials that did not show a beneficial effects of immediate radical treatments on overall survival (OS),^9,10,11^ even after 15 years from diagnosis.^12^ Nonetheless, the adoption of active surveillance is still heterogenous, both among and within countries.^13,14,15,16,17,18,19,20,21^ In Italy, the main evidence on active surveillance is from the Prostate cancer Research International (PRIAS) study, an international cohort study, including 16 Italian centers, that enrolled highly selected patients who chose active surveillance at diagnosis.^22^

Up to 2015, in the Regional Oncology Network (RON) of the Piemonte and Valle d’Aosta regions in Northern Italy (approximate population, 4.5 million), few, highly selected patients were offered active surveillance, despite local guidelines recommending active surveillance for LRPC, as reported by a nonsystematic survey among chiefs of urology and radiation oncology units.^23^ Several factors were found to act as barriers to active surveillance, including cultural, medicolegal, and psychological factors, both among patients and physicians, as reported by others.^24,25,26,27,28,29,30,31^ To overcome these barriers and offer active surveillance to all patients for whom this option was suitable, we launched a population-based cohort study (Sorveglianza attiva o trattamento radicale alla diagnosi per tumori della prostata a basso rischio [START]) in 2015, involving all urology and radiation oncology centers of the RON. The aim of the study was to evaluate acceptability, safety, and costs of active surveillance compared with immediate active radical treatments in well-informed patients with newly diagnosed LRPC. In this study, we report the overall acceptance of active surveillance, the factors associated with patient’s choice of initial management, and the early clinical outcomes.

## Methods

This cohort study was approved by all regional ethics committees. Each participant received both verbal and written information on available treatments and on study participation and subsequently signed a written informed consent form before enrollment in the study. This study followed the Strengthening the Reporting of Observational Studies in Epidemiology (STROBE) reporting guideline for cohort studies.

### Study Design

The START protocol was developed by a multidisciplinary panel of specialists of the RON, including urologists, radiation oncologists, pathologists, oncologists, epidemiologists, and patients’ representatives and has been registered at ClinicalTrials.gov (identifier: NCT03348722). All the RON public hospital units of urology, radiotherapy, and pathology were involved and actively participated to the study.

All patients with newly diagnosed PC fulfilling the low-risk definition and living in Piemonte or Valle D’Aosta, Italy, were eligible. Patients received verbal and written information about their diagnosis and prognosis, together with an information leaflet, written in plain language with the involvement of previous patients with PC, describing benefits and risks of the available management strategies, including radical prostatectomy (RP), radiation therapy (RT) or other local treatments, and active surveillance, to allow for an informed choice.

### Inclusion and Exclusion Criteria

The main eligibility criteria for LRPC were similar to those of the PRIAS study,^22^ namely, no contraindication to radical treatments, clinical stage T1c or T2a, PSA levels of 10 ng/mL or less (to convert to micrograms per liter, multiply by 1), and a Gleason Pattern Score (GS) of 3 + 3 (GS 3 + 4 allowed in men aged >70 years.). The maximum number of positive cores was accorded to the number of random biopsies performed and to the execution of multiparametric magnetic resonance imaging (MRI). Detailed inclusion and exclusion criteria are reported in eTable 1 in Supplement 1.

Specialists of each pathology unit had the option to ask for an independent, centralized biopsy specimen review at diagnosis to improve the interpretation of the borderline diagnoses, considering the modified GS. The reviews were performed by a group of 2 to 4 external uropathologists (with 2 permanent members and 2 randomly selected from the regional pathologist group) via a web-based platform in which scanned slide images were uploaded without the initial diagnosis of the local pathologist.

### Management Strategies and Follow-Up

Patients accepting active surveillance were offered a structured follow-up program, with scheduled appointments for repeating PSA testing, clinical assessments, and a repeated biopsies at 12 and 48 months (eTable 2 in Supplement 1). Patients without PSA variations or other clinical warnings during follow-up could undergo multiparametric MRI instead of biopsy at 48 months. Patients in active surveillance could switch to active treatment at any time, depending on patient’s choice, or if they were recommended to do so because of worsening of clinical parameters (eg, GS, stage, increasing PSA) (eTable 3 in Supplement 1).

For patients choosing active treatments, the follow-up schedule was similar to that of active surveillance, but with clinical assessments and PSA measurements every 6 months and no planned rebiopsy.

### Data Collection

A dedicated website was set up with public and reserved areas for data collection. Baseline clinical, histological, and psychological data (Hospital Anxiety and Depression Scale and Multidimensional Health Locus of Control Scale); details on any treatment received; and follow-up data were prospectively collected by the local clinical team and centrally verified and uploaded to the START database by dedicated data managers.

### Study Size

Considering the increasing trend of PC incidence and an expected proportion of 25% of LRPC, the protocol sample size was calculated to reach approximately 750 patients in active surveillance within 3 years of accrual. However, according to more recent data of the Piemonte Cancer Registry and a more accurate estimate of the proportion of LRPC fulfilling all the inclusion criteria and accepting to participate in START, the number of patients enrolled was lower, corresponding to approximately 5% of the total incidence in the population. Therefore, the study protocol was amended to extend the enrollment up to 6.5 years to reach the expected sample size of the active surveillance cohort.

### Statistical Analysis

Patient and physician factors associated with different choices of management were analyzed with a multilevel logistic regression model (level 1, patient characteristics; level 2, center that enrolled the patient) to account for the clustering of data within centers. To screen the baseline variables to be estimated in the final model, a backward stepwise selection strategy was applied, with large statistical thresholds to include *P* = .50 and to retain *P* = .25 variables.

The survival status for all enrolled patients was systematically checked at the end of March 2023 through the regional population register. Causes of death were obtained from death certificates. OS was analyzed for the entire cohort according to an intention-to-treat approach. Active surveillance and active treatments groups were compared with the Kaplan-Meier method and with a Cox multivariable model (adjusting for age, comorbidity, GS, and number of biopsy cores positive for PC, with a GS of at least 3 + 3). Treatment-free survival (TFS), ie, the proportion of patients in active surveillance program alive and not undergoing active treatment for PC during follow-up, was estimated with the Kaplan-Meier method. According to the study protocol, all comparisons were made between active surveillance and active treatments; however, considering the substantial differences between patients who received RP vs RT, the same comparisons have been repeated between active surveillance and RP groups.

All statistical tests were 2-sided and 95% CIs were estimated for all outcome measures (odds ratios [OR] and hazard ratios [HR]). Data were analyzed with SAS statistical software version 14.1 (SAS Institute). Data were analyzed from January to May 2023.

## Results

**Figure 1.**
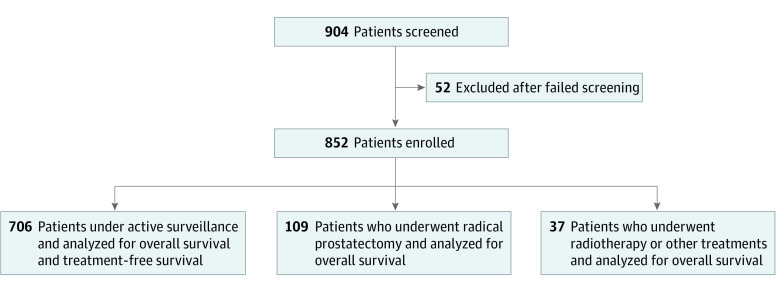
Patient Enrollment Flowchart Other treatments included high-intensity focused ultrasound.

From June 2015 to December 2021, 904 male patients were enrolled and 852 patients (median [IQR] age, 70 [64-74] years) were included in analyses; 52 patients were excluded because of screening failure. The patient recruitment flowchart is presented in Figure 1. After reading the information leaflet on the treatment options for LRPC and an in-depth discussion with the specialists, 706 patients (82.9%) chose active surveillance, 109 patients (12.8%) chose RP, and 37 patients (4.3%) chose RT or other treatments (30 patients received RT; 7 patients received high-intensity focused ultrasound) as first management strategy (Figure 1).

Some heterogeneity was observed among the participating centers, both in the number of patients enrolled (only partially explained by the patient load of each center) and in the proportion of patients who chose active surveillance. Among 18 urology units that performed approximately 95% of the total prostatectomies in the RON, the median (IQR) ratio between the total number of patients who underwent RP for prostate cancer (any stage) and the number of those enrolled in START who chose active surveillance was 10.4 (7.1-12.6), and the proportion of patients choosing active surveillance ranged between 38% and 100%.

The clinical, sociodemographic, and psychological characteristics of patients at diagnosis are summarized in Table 1. Patients who chose RP were younger and had fewer comorbidities than those in active surveillance or RT and other treatments. Patients in active surveillance had a lower risk profile for PC, with lower baseline PSA values, fewer positive biopsy cores, lower clinical stage, and lower GS than patients in the RP or RT and other treatments groups (Table 1). A higher proportion of patients whose biopsy was centrally reviewed and of those evaluated by a multidisciplinary team chose active surveillance (Table 1). During the study period, there was an upward trend in the choice of active surveillance, from 77.7% of patients in 2015 to 2017 to 90.2% of patients in 2020 to 2021 (Table 1).

**Table 1.** Patients’ Characteristics at Baseline by Initial Treatment Choice

Characteristic	Patients, No. (%)		
	Active surveillance (n = 706)	Radical prostatectomy (n = 109)	Radiotherapy or HIFU (n = 37)
Age, y			
≤64	174 (24.6)	33 (30.3)	6 (16.2)
65-69	165 (23.4)	23 (21.1)	4 (10.8)
70-74	205 (29.0)	41 (37.6)	18 (48.6)
≥75	162 (22.9)	12 (11.0)	9 (24.3)
Charlson Comorbidity Index			
0	380 (53.8)	61 (56.0)	21 (56.8)
1	142 (20.1)	32 (29.4)	8 (21.6)
≥2	131 (18.6)	11 (10.1)	7 (18.9)
NA	53 (7.5)	5 (4.6)	1 (2.7)
PSA, ng/mL^c^			
≤7	558 (79.0)	79 (72.5)	28 (75.7)
8-10	148 (21.0)	30 (27.5)	9 (24.3)
Sampling technique			
Random or saturation	504 (71.4)	70 (64.2)	27 (73.0)
Target with or without random	202 (28.6)	39 (35.8)	10 (27.0)
Positive biopsy cores, No.			
1	535 (75.8)	74 (67.9)	22 (59.5)
2	171 (24.2)	35 (32.1)	15 (40.5)
First biopsy revision			
No	547 (77.5)	94 (86.2)	33 (89.2)
Yes	159 (22.5)	15 (13.8)	4 (10.8)
Clinical stage			
T1c	601 (85.1)	83 (76.1)	28 (75.7)
T2a	105 (14.9)	26 (23.9)	9 (24.3)
Gleason Score			
3 + 3	599 (84.8)	78 (71.6)	18 (48.6)
3 + 4	107 (15.2)	31 (28.4)	19 (51.4)
Magnetic resonance imaging			
No	390 (55.2)	51 (46.8)	21 (56.8)
Yes	316 (44.8)	58 (53.2)	16 (43.2)
Multidisciplinary assessment			
No	507 (71.8)	96 (88.1)	35 (94.6)
Yes	199 (28.2)	13 (11.9)	2 (5.4)
Enrolling unit			
Radiation oncology	52 (7.4)	0 (0.0)	15 (40.5)
Urology	654 (92.6)	109 (100.0)	22 (59.5)
Year of diagnosis			
2015-2017	258 (77.7)	56 (16.9)	18 (5.4)
2018-2019	274 (83.8)	40 (12.2)	13 (4.0)
2020-2021	174 (90.2)	13 (6.7)	6 (3.2)
Employment status			
Unemployed or retired	384 (54.4)	55 (50.5)	22 (59.5)
Employed	99 (14.0)	20 (18.3)	1 (2.7)
NA	223 (31.6)	34 (31.2)	14 (37.8)
Education, years			
≤7	98 (13.9)	15 (13.8)	7 (18.9)
8-13	179 (25.4)	31 (28.4)	9 (24.3)
14	153 (21.7)	26 (23.9)	5 (13.5)
NA	276 (39.1)	37 (33.9)	16 (43.2)
Living with other people			
No	47 (6.7)	5 (4.6)	1 (2.7)
Yes (partner)	384 (54.4)	64 (58.7)	17 (45.9)
Yes (others)	44 (6.2)	5 (4.6)	3 (8.1)
NA	231 (32.7)	35 (32.1)	16 (43.2)
Anxiety			
No	434 (61.5)	70 (64.2)	18 (48.6)
Borderline or high	121 (17.1)	17 (15.6)	9 (24.3)
NA	151 (21.4)	22 (20.2)	10 (27.0)
Depression			
No	505 (71.5)	81 (74.3)	26 (70.3)
Borderline or high	50 (7.1)	6 (5.5)	1 (2.7)
NA	151 (21.4)	22 (20.2)	10 (27.0)
Self-reliance			
Low	137 (19.4)	18 (16.5)	10 (27.0)
Intermediate	228 (32.3)	40 (36.7)	10 (27.0)
High	148 (21.0)	17 (15.6)	6 (16.2)
NA	193 (27.3)	34 (31.2)	11 (29.7)
Reliance on chance or luck			
Low	136 (19.3)	19 (17.4)	6 (16.2)
Intermediate	232 (32.9)	42 (38.5)	18 (48.6)
High	146 (20.7)	15 (13.8)	2 (5.4)
NA	192 (27.2)	33 (30.3)	11 (29.7)
Trust in physicians			
Low	164 (23.2)	35 (32.1)	11 (29.7)
Intermediate	179 (25.4)	20 (18.3)	5 (13.5)
High	172 (24.4)	20 (18.3)	10 (27.0)
NA	191 (27.1)	34 (31.2)	11 (29.7)
Trust in other people			
Low	144 (20.4)	25 (22.9)	7 (18.9)
Intermediate	222 (31.4)	32 (29.4)	15 (40.5)
High	146 (20.7)	19 (17.4)	4 (10.8)
NA	194 (27.5)	33 (30.3)	11 (29.7)

Abbreviations: HIFU, high-intensity focused ultrasound; NA, not available; PSA, prostate specific antigen.

SI conversion factor: To convert PSA to micrograms per liter, multiply by 1.

Some differences between groups in terms of employment status, education, and household characteristics were expected, as they reflect differences in age distribution. A few differences between groups were also detected by the Hospital Anxiety and Depression Scale and the Multidimensional Health Locus of Control Scale questionnaires, with a tendency toward higher self-reliance, reliance on physicians, reliance on chance, and trust in other people among patients who chose active surveillance compared with those opting for RP (Table 1).

### Factors Associated to the Choice of Active Surveillance

Table 2 reports the results of the multilevel logistic regression models, considering centers as random effects, to evaluate factors associated with the choice of active surveillance compared with any radical treatment and compared with RP as first management option. Compared with patients who chose any radical treatments, patients who chose active surveillance were older (eg, age ≥75 vs ≤64 years: OR, 4.27; 95% CI, 1.98-9.22) and had a higher Charlson Comorbidity Index (≥2 vs 0: OR, 1.98; 95% CI, 1.02-3.85). Worse prostate cancer prognostic factors, such as stage T2a (OR, 0.54; 95% CI, 0.31-0.94) and GS 3 + 4 (OR, 0.20; 95% CI, 0.11-0.37) were associated with lower odds of choosing active surveillance over any active treatment. An independent revision of the prostate biopsy specimen (OR, 2.35; 95% CI, 1.26-4.38) and a multidisciplinary assessment (OR, 2.65; 95% CI, 1.38-5.11) were associated with choosing active surveillance rather than any active treatment. During 6.5 years, the proportion of patients who chose active surveillance increased up to 90% (OR per year, 1.30; 95% CI, 1.13-1.49). No other sociodemographic, psychological, or clinical characteristics showed meaningful associations with initial treatment choice after adjustment for other variables, suggesting a preponderance of urologic clinical judgment over other patient characteristics in this choice. The results of the comparison between active surveillance vs radical prostatectomy were similar, even with some loss of precision due to the reduced sample size (Table 2). After the adjustment for unbalanced patient characteristics at baseline, the covariance parameter estimate of the centers, included as random effects, was 0.75 (*P* = .006), which can be approximated to an intraclass correlation coefficient of 18.6%, confirming a relevant association of the center with patients’ choice.

**Table 2.** Associations of Patient Characteristics at Baseline With Initial Treatment Choice

Characteristic	Active surveillance vs any radical treatment		Active surveillance vs radical prostatectomy	
	OR (95%CI)	*P* value	OR (95%CI)	*P* value
Age group				
<65	1 [Reference]	NA	1 [Reference]	NA
65-69	1.39 (0.75-2.56)	.29	1.38 (0.72-2.63)	.33
70-74	1.29 (0.70-2.39)	.41	1.35 (0.69-2.65)	.38
≥75	4.27 (1.98-9.22)	<.001	5.67 (2.31-13.96)	<.001
Charlson Comorbidity Index				
0	1 [Reference]	NA	1 [Reference]	NA
1	0.72 (0.43-1.20)	.20	0.65 (0.38-1.13)	.13
≥2	1.98 (1.02-3.85)	.04	2.05 (0.95-4.44)	.07
NA	2.56 (0.87-7.55)	.09	2.05 (0.66-6.39)	.21
Reliance on chance or luck				
Low	1 [Reference]	NA	1 [Reference]	NA
Intermediate or NA	0.67 (0.38-1.20)	.17	0.68 (0.36-1.30)	.24
High	1.36 (0.63-2.92)	.42	1.11 (0.49-2.53)	.80
Trust in physicians				
Low	1 [Reference]	NA	1 [Reference]	NA
Intermediate or NA	1.44 (0.85-2.44)	.17	1.35 (0.76-2.39)	.30
High	1.52 (0.83-2.78)	.17	1.77 (0.89-3.50)	.10
PSA, ng/mL				
<8	1 [Reference]	NA	1 [Reference]	NA
8-10	0.75 (0.46-1.23)	.25	0.77 (0.45-1.33)	.34
No. of positive cores				
1	1 [Reference]	NA	1 [Reference]	NA
2	0.69 (0.43-1.11)	.13	0.72 (0.43-1.22)	.22
First biopsy revision				
No	1 [Reference]	NA	1 [Reference]	NA
Yes	2.35 (1.26-4.38)	.009	2.33 (1.18-4.58)	.02
Stage				
T1c	1 [Reference]	NA	1 [Reference]	NA
T2a	0.54 (0.31-0.94)	.03	0.51 (0.28-0.93)	.03
Gleason score				
3 + 3	1 [Reference]	NA	1 [Reference]	NA
3 + 4	0.20 (0.11-0.37)	<.001	0.23 (0.11-0.47)	<.001
Multidisciplinary assessment				
No	1 [Reference]	NA	1 [Reference]	NA
Yes	2.65 (1.38-5.11)	.005	2.36 (1.17-4.76)	.02
Year of diagnosis				
2015-2017	1 [Reference]	NA	1 [Reference]	NA
2018-2019	1.70 (0.95-3.07)	.07	1.61 (0.84-3.10)	.13
2020-2021	3.81 (1.72-8.42)	.005	3.91 (1.56-9.77)	.01

Abbreviations: NA, not applicable; OR, adjusted odds ratio; PSA, prostate specific antigen.

SI conversion factor: To convert PSA to micrograms per liter, multiply by 1.

### Factors Associated With OS

**Figure 2.**
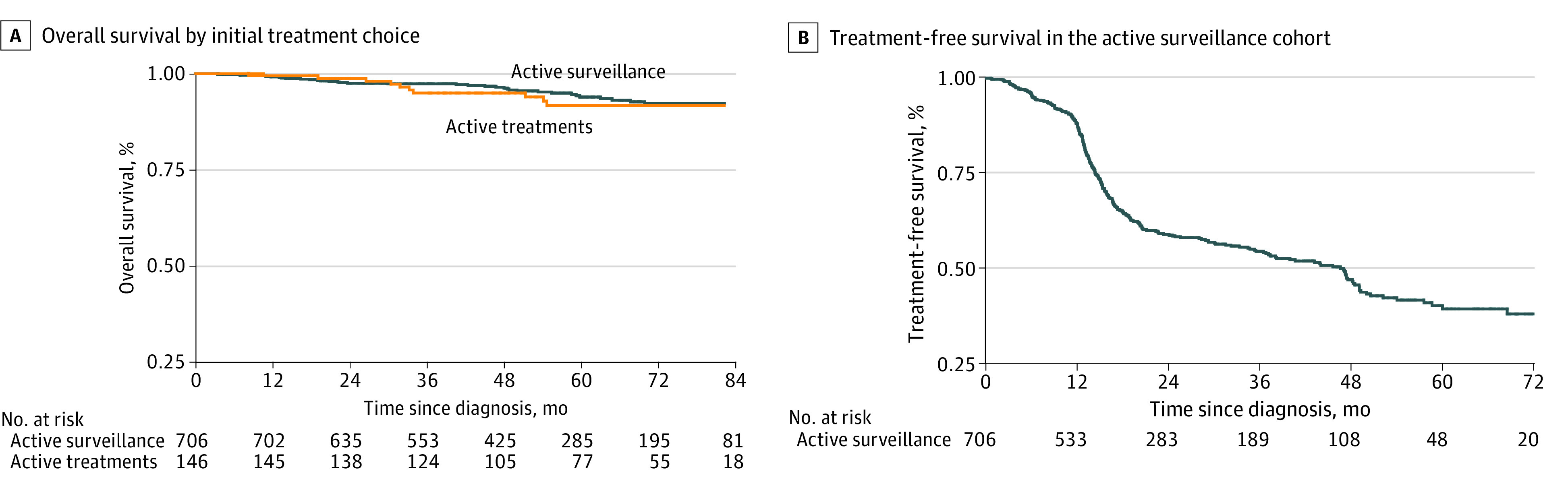
Overall Survival by Initial Treatment Choice and Treatment-Free Survival in the Active Surveillance Cohort

After a median (IQR) follow-up of 57 (41-76) months, with no loss at follow-up, a total of 46 patients had died, including 3 who died of PC. Of 706 patients in active surveillance, 36 (5.1%) died (1 patient died of PC); of 109 patients who underwent RP, 6 (5.5%) died (1 patient died of PC), and of 37 patients who received RT or other treatments, 4 (10.8%) died (1 patient died of PC). The Kaplan-Meier curves of OS by initial treatment choice are presented in Figure 2A. The 5-year OS of the entire cohort was 94.2% (95% CI, 92.1%-95.8%), without significant difference between patients in active surveillance (94.8%; 95% CI, 92.6%-96.4%) and those actively treated at diagnosis (91.7%; 95% CI, 84.9%-95.5%). The adjusted associations between the initial choice and OS are reported in Table 3. Active surveillance was not associated with OS compared with any radical treatment (HR, 0.86; 95% CI, 0.41-1.79), nor compared with radical prostatectomy (HR, 0.90; 95% CI, 0.37-2.20). In both comparisons, older age and a Charlson Comorbidity Index of 2 or greater were negatively associated with OS.

**Table 3.** Adjusted Associations of Active Surveillance vs Any Radical Treatment and vs Radical Prostatectomy With Overall Survival

Characteristic	Active surveillance vs any radical treatment		Active surveillance vs radical prostatectomy	
	aHR (95% CI)	*P* value	aHR (95% CI)	*P* value
Active surveillance	0.86 (0.41-1.79)	.68	0.90 (0.37-2.20)	.82
Age, per 1-y increase	1.07 (1.01-1.13)	.02	1.07 (1.01-1.13)	.02
Charlson Comorbidity Index				
0	1 [Reference]	NA	1 [Reference]	NA
1	1.04 (0.47-2.33)	.92	1.05 (0.45-2.46)	.92
≥2	2.51 (1.29-4.87)	.007	2.70 (1.34-5.41)	.005
Positive biopsy cores				
1	1 [Reference]	NA	1 [Reference]	NA
2	1.35 (0.73-2.48)	.34	1.33 (0.70-2.53)	.38
Gleason score				
3 + 3	1 [Reference]	NA	1 [Reference]	NA
3 + 4	1.68 (0.86-3.30)	.13	1.42 (0.69-2.93)	.35

Abbreviations: aHR, adjusted hazard ratio; NA, not applicable.

### TFS for Patients in Active Surveillance

The Kaplan-Meier curve for TFS of patients in active surveillance is shown in Figure 2B. During follow-up, 297 patients (42.1%) starting active surveillance shifted to an active treatment and 67 patients (9.5%) were lost to follow-up while in active surveillance. At 12 months, the TFS rate was 87.8% (95% CI, 85.0%-90.1%), then this percentage showed a remarkable reduction until 24 months (59.0%; 95% CI, 54.8%-62.9%) and thereafter remained stable (36 months: 54.5%; 95% CI, 50.2%-58.6%; 48 months: 47.0%; 95% CI, 42.2%-51.7%). The most frequent reasons of abandoning active surveillance reported by physicians were biochemical progression (143 patients [48.2%]), upstaging or upgrading (59 patients [19.9%]), patient decision (54 patients [18.2%]), and doctor decision (10 patients [3.4%]). The treatments most frequently chosen by patients who ceased active surveillance were RP (170 patients [57.2%]) and RT with or without hormone therapy (102 patients [34.3%]).

## Discussion

The START cohort study was designed as a population-based cohort study with both research and intervention purposes. With detailed, prospective data collection, we assessed patients’ treatment preferences at the time of diagnosis and patients’ retention in active surveillance and compared clinical outcomes between groups of patients according to their initial choice. Furthermore, the research context provided a useful strategy to promote the regional implementation of active surveillance for patients with localized LRPC through careful and balanced information provided by the physician, overcoming long-standing cultural and organizational barriers.

The START study provides valuable evidence, given that most of the available studies in this setting are retrospective, with cross-sectional designs or with record linkages between registries and databases, and most studies with a prospective design are cohorts of selected patients in active surveillance only, monocentric, or from selected centers.^8^

The main remarkable finding of START is represented by the widespread adoption of active surveillance in our RON since the beginning of the study, and the increasing trend over time, reaching approximately 90% of eligible patients in 2020 to 2021. This dramatic change was likely attributable to the START study, as in 2009, the local community of specialists and researchers had already developed a regional guideline on prostate cancer with a recommendation to support active surveillance that remained mostly unattended until the beginning of this study.

According to our findings, clinical judgment was the main driver associated with patients’ choice, rather than psychological or sociocultural issues. General health status (older age, with associated comorbidities) was considered as a partial contraindication to RP, whereas worse clinical prognostic factors, such as higher tumor stage or GS 3 + 4, were associated with lower odds of choosing active surveillance. In addition, some factors reflecting shared decisions among specialists (biopsy revision, multidisciplinary discussion) were positively associated with the choice of active surveillance, suggesting again the crucial role of the health care organization and of the treating physicians in guiding patients’ choices.^32,33^ Along a similar vein, we also highlight the significant heterogeneity among centers in the proportion of patients who received active surveillance, in line with previous experiences.^13,16,20,34^

Noteworthy, none of the patient-related factors (ie, education, occupation, civil status, anxiety, depression, or dimensions of the Multidimensional Health Locus of Control Scale questionnaire) were associated with initial choice of treatment in our adjusted analyses, confirming the substantial equity of access to care in the Italian National Health Service. Our results are in line with several studies previously published suggesting that a physician recommendation for active surveillance is the factor with the strongest role in patient decision-making.^25,29,35,36^

To counterbalance the success of the large regional adoption of active surveillance, we must acknowledge that the dropout rate of patients in active surveillance was high, especially between 12 and 24 months after diagnosis. The reasons of this early abandoning of active surveillance will be further investigated, but the role of the 12 months rebiopsy, especially among patients who also underwent MRI, the reevaluation of clinical parameters, and the influence of centers with different degrees of confidence in active surveillance, were likely the most relevant factors in the choice to end active surveillance. Biochemical progression played a critical role in determining the switch to active treatment, being the reason for active surveillance abandonment in more than 50% of patients. The comparison with similar experiences in the literature is limited; other studies based on single- or multi-institutional nonrandomized cohorts generally show a lower dropout rate, with TFS rates between 48% and 76% at 5 years.^5^

A limiting factor in choosing active surveillance, for both patients and physicians, is the fear of disease progression and, ultimately, death. Data available from literature on long-term OS are reassuring,^11^ but excesses in incidence of metastases and in cancer-specific deaths were reported for patients randomized to active monitoring in the PROTECT trial, the largest available randomized trial.^9,12,37^ In our study, the OS was not worse in patients who initially chose active surveillance instead of a radical treatment in an adjusted, intention-to-treat analysis. This result was confirmed when limiting the comparison of OS between active surveillance and RP cohorts. Considering the high proportion of patients who abandoned active surveillance during the second year of follow-up, an extended follow-up is necessary to assess long-term outcomes.

### Limitations

The main limitations of this study are the variability among centers, both in enrolling patients (with the possible loss of some eligible patients) and in shaping their choices, and the high rate of patients who abandoned active surveillance during the second year of follow-up. Another limitation of this study is the involvement of all regional centers treating patients with PC, including those with limited resources and experience in data collection and clinical research, which increased heterogeneity among centers. Furthermore, given the relatively short duration of follow-up, prolonged observation of the entire cohort will be conducted to enable long-term comparison of OS, quality of life, and costs. In the meantime, periodic meetings will be held to consolidate results and for further improvements.

## Conclusions

The START cohort study had 2 main objectives: to promote the implementation of active surveillance in the entire oncology network of 2 regions in Northern Italy and to understand the acceptability, determinants, and the outcomes associated with active surveillance vs radical treatments in a comparative effectiveness framework. The first objective has been achieved with results beyond any expectations, considering the participation of almost all urology and radiation oncology units and the crucial support of the pathology departments in reviewing the first biopsies. The START cohort study represents a valuable contribution to evidence on active surveillance and an example of how pragmatic research, embedded in clinical practice, can promote health care quality improvements.

## Notes

Supplement 1. **eTable 1.** Inclusion and Exclusion Criteria **eTable 2.** Calendar of Controls During the First 5 Years of Patient Follow-Up **eTable 3.** Criteria for Continuation of AS in the START Study

Supplement 2.
Members of the START Collaborative Group

Supplement 3.
Data Sharing Statement
