# Supplementary material for: Patient and Context Factors in the Adoption of Active Surveillance for Low-Risk Prostate Cancer
Source: JAMA Netw Open. 2023 Oct 17;6(10):e2338039. doi: 10.1001/jamanetworkopen.2023.38039 (PMC10582795; doi:10.1001/jamanetworkopen.2023.38039)
Supplement: Supplement 1. — eTable 1. Inclusion and Exclusion Criteria eTable 2. Calendar of Controls During the First 5 Years of Patient Follow-Up eTable 3. Criteria for Continuation of AS in the START Study [file jamanetwopen-e2338039-s001.pdf]

Supplemental Online Content

Ciccone G, De Luca S, Oderda M, et al; for the START Collaborative Group. Patient and context factors in the adoption of active surveillance for low-risk prostate cancer. *JAMA Netw Open*. 2023;6(10):e2338039. doi:10.1001/jamanetworkopen.2023.38039

eTable 1. Inclusion and Exclusion Criteria
eTable 2. Calendar of Controls During the First 5 Years of Patient Follow-Up
eTable 3. Criteria for Continuation of AS in the START Study

This supplemental material has been provided by the authors to give readers additional information about their work.

© 2023 Ciccone G et al. *JAMA Network Open.*

eTable 1. Inclusion and exclusion criteria

Inclusion Criteria:

1. Newly diagnosed low risk prostate cancer patients, defined according to the presence of all the following criteria:
    - diagnosis of adenocarcinoma of the prostate
    - prostate cancer clinical stage T1c or T2a
    - PSA <=10ng/ml at diagnosis
    - adequate biopsy sampling according to prostate volume
    - maximum number of positive biopsy cores for prostate adenocarcinoma:
        - 2, in case of biopsy with random sampling and less than 20 samples
        - 3, in case of biopsy with random sampling and 20-26 samples
        - 4, in case of biopsy with random sampling and more than 26 samples
        - If a multi-parametric MRI of the prostate was performed at the time of diagnosis and multiple samples were taken from each target lesion, two or more positive samples from the same target lesion (regardless of the percentage of disease present in the samples) must also be considered as one positive core for the purpose of calculating the total number of positive cores. The number of positive biopsy cores will therefore be calculated as the sum of the positive target lesions + any positive random samples; the total number of samples will be calculated as the sum of the number of random samples and the number of biopsied target lesions.
    - Gleason grade 3+3 (in patients aged>70 Gleason 3+4)
2. Residence in Piemonte or Valle D'Aosta regions;
3. Patients suitable for radical treatment (surgery or radiotherapy/HIFU);
4. Age at diagnosis <= 75 years or >75 years if frailty assessment (measured with the G8 score)> = 14;
5. Patients' suitability for expressing a valid consent to participate in the study.

Exclusion Criteria:

1. Patients previously treated for prostate cancer
2. Patients not willing to undergo radical treatments

© 2023 Ciccone G et al. *JAMA Network Open.*

eTable 2. Calendar of controls during the first 5 years of patient follow-up (adapted from PRIAS). (For patients undergoing radical treatments, the monitoring schedule is indicative, but must be adhered to at least at six-monthly intervals during the first 5 years)

Year	1					2				3		4		5	
Month	0*	3	6	9	12	15	18	21	24	30	36	42	48	54	60
PSA test	✓	✓	✓	✓	✓	✓	✓	✓	✓	✓	✓	✓	✓	✓	✓
DRE	✓		✓		✓		✓		✓		✓		✓		✓
Biopsy**	✓				✓								✓		
Clinical examination	✓		✓		✓		✓		✓		✓		✓		✓
QoL questionnaires	✓		✓		✓		✓		✓		✓		✓		✓

* Diagnosis
** For patients in active surveillance only: a biopsy could be repeated at 7 and 10 years, and every 5 years thereafter.
If PSA doubling time <=10 years: repeat biopsy (if not performed within the last year); as an alternative to biopsy, multi-parametric MRI of the prostate, possibly followed by targeted biopsy of suspicious lesions

eTable 3. Criteria for continuation of AS in the START study (adapted from PRIAS)
The following criteria are a useful guide to re-evaluate the decision with the patient in AS during follow-up. For patients that are no more suitable for radical treatments (for age, comorbidity, or other reasons) consider and discuss the possibility of a de-escalation of the follow-up schedule towards a watchful waiting regimen.

1) Clinical:
   - Clinical stage < = T2a (reassess the patient's overall risk if clinical stage = T2b o T2c)
2) Istological (Gleason Score according to ISUP 2005):
   - Gleason-score 3+3=6 or less *(Gleason score 3+4 allowed for patients aged >=70 years)*
   - No more than one or two positive biopsy cores (considering specimens from the same mpMRI target lesion as a single core)
3) Biochemical:
   - A single altered PSA value should be repeated after a few weeks of treatment of possible infectious diseases or other non-neoplastic causes
   - PSA doubling time (PSA DT) > 10 years
   - If PSA DT <=10 years: repeat biopsy (if not performed within the last year); as an alternative to biopsy, multi-parametric MRI of the prostate, possibly followed by targeted biopsy of suspicious lesions
   - If PSA > 20 ng/ml: perform a bone scintigraphy
4) Personal:
   - Patient satisfied to continue AS

© 2023 Ciccone G et al. *JAMA Network Open.*
