# Supplementary material for: Patient and Context Factors in the Adoption of Active Surveillance for Low-Risk Prostate Cancer
Source: JAMA Netw Open. 2023 Oct 17;6(10):e2338039. doi: 10.1001/jamanetworkopen.2023.38039 (PMC10582795; doi:10.1001/jamanetworkopen.2023.38039)
Supplement: Supplement 2. — Members of the START Collaborative Group [file jamanetwopen-e2338039-s002.pdf]

Supplemental Online Content: Nonauthor Collaborators
*First name, last name, and suffix (if applicable) are required and will appear in PubMed.

*Group Name(s): The START Collaborative Group							
*First Name and Middle Initial(s)	*Last Name	*Suffix (eg, Jr, III)	Academic Degrees	Institution	Location (city, state/province, country)	Role or Contribution, eg, chair, principal investigator	Group (if more than 1 Group listed in the byline) and/or Subgroup (eg, Steering Committee)
Manuela	Alessio		MSc, PhD	SS Progetti, Ricerca e Innovazione, Ospedale Michele e Pietr	Verduno (Cuneo), Italy	Data collection and assembly	
Scipio	Annoscia		MD	Urologia, Ospedali Riuniti ASL TO4	Ivrea, Ciriè, Chivasso (Torin	Steering committee	
Daniela	Antonini		MD	Anatomia Patologica, PO Martini	Torino, Italy	Biopsy review	
Marisa	Arrondini		MD	Anatomia Patologica, AOU Maggiore della Carità e Università	Novara, Italy	Biopsy review	
Emanuele	Baldassarre		MD	Urologia, PO Umberto Parini	Aosta, Italy	Provision of patients	
Paola	Barbieri		MD	Anatomia Patologica, AO SS Antonio e Biagio e Cesare Arrigo	Alessandria, Italy	Biopsy review	
Franco	Bardari		MD	Urologia, PO San Giovanni Bosco	Torino, Italy	Provision of patients	
Gaetano	Belli		MD	Radioterapia, Istituto di Candiolo, Fondazione del Piemonte	Candiolo (Torino), Italy	Provision of patients	
Maurizio	Bellina		MD	Urologia, PO Rivoli	Rivoli (Torino), Italy	Provision of patients	
Donata	Bellis		MD	Anatomia Patologica, PO Nuovo Ospedale degli Infermi	Ponderano (Biella), Italy	Biopsy review	
Fabio	Bonini		MD	Urologia, PO San Giacomo	Novi Ligure (Alessandria), I	Provision of patients	
Giulio	Bonvissuto		MD	Urologia, PO Rivoli	Rivoli (Torino), Italy	Provision of patients	
Martino	Bosco		MD	Anatomia Patologica, PO Michele e Pietro Ferrero	Verduno (Cuneo), Italy	Biopsy review	
Francesco	Bracco		MD	Urologia, AOU Città della Salute e della Scienza e Università d	Torino, Italy	Provision of patients	
Rodolfo	Brizio		MD	Anatomia Patologica, AO Santa Croce e Carle	Cuneo, Italy	Biopsy review	
Francesco	Brunetti		HSD	Epidemiologia Clinica e Valutativa, AOU Città della Salute e d	Torino, Italy	Web site manager	
Andrea	Buffardi		MD	Urologia, PO San Giovanni Bosco	Torino, Italy	Provision of patients	
Silvia	Cagnasso		HSD	Urologia, PO Michele e Pietro Ferrero	Verduno (Cuneo), Italy	Data collection and assembly	
Eugenio	Cagnazzi		MD	Urologia, PO Pinerolo	Rivoli (Torino), Italy	Provision of patients	
Giorgio	Calleris		MD	Urologia, AOU Città della Salute e della Scienza e Università d	Torino, Italy	Provision of patients	
Paola	Campisi		MD	Anatomia Patologica, AO Ordine Mauriziano	Torino, Italy	Biopsy review	
Laura	Caramanico		MD	Anatomia Patologica, PO Umberto Parini	Aosta, Italy	Biopsy review	
Mariateresa	Carchedi		MD	Urologia, PO Rivoli	Rivoli (Torino), Italy	Provision of patients	
Ugo	Casalone		HSD	Epidemiologia Clinica e Valutativa, AOU Città della Salute e d	Torino, Italy	web-site, e-CRF	
Paola	Cassoni		MD, PhD	Anatomia Patologica 2, AOU Città della Salute e della Scienza	Torino, Italy	Biopsy review	
Manuela	Ceccarelli		MD	Direzione Sanitaria, AOU Città della Salute e della Scienza di T	Torino, Italy	Steering committee	
Germano	Chiapello		MD	Urologia, AO Santa Croce e Carle	Cuneo, Italy	Provision of patients	
Elena	Cianini		MD	Urologia, PO Nuovo Ospedale degli Infermi	Ponderano (Biella), Italy	Provision of patients	
Francesca	Clot		MSc	Radioterapia, AOU Città della Salute e della Scienza e Univers	Torino, Italy	Data collection and assembly	
Nicola	Cruciano		MD	Urologia, PO Maria Vittoria	Torino, Italy	Provision of patients	
Michele	Cussotto		MD	Urologia, PO Cardinal Massaia	Asti, Italy	Provision of patients	
Paolo	De Angelis		MD	Urologia, AOU Maggiore della Carità e Università del Piemon	Novara, Italy	Provision of patients	
Paolo	De Giuli		MD	Anatomia Patologica, PO Michele e Pietro Ferrero V	Verduno (Cuneo), Italy	Biopsy review	

Supplemental Online Content: Nonauthor Collaborators
*First name, last name, and suffix (if applicable) are required and will appear in PubMed.

*First Name and Middle Initial(s)	*Last Name	*Suffix (eg, Jr, III)	Academic Degrees	Institution	Location (city, state/province, country)	Role or Contribution, eg, chair, principal investigator	Group (if more than 1 Group listed in the byline) and/or Subgroup (eg, Steering Committee)
Elena	Delmastro		MD	Radioterapia, Istituto di Candiolo - Fondazione del Piemonte	Candiolo (Torino), Italy	Provision of patients	
Luisa	Delsedime		MD	Anatomia Patologica 2, AOU Città della Salute e della Scienza	Torino, Italy	Biopsy review	
Jessica	Di Martino		MD	Urologia, AOU Maggiore della Carità e Università del Piemon	Novara, Italy	Provision of patients	
Natalia	Dogliani		MD	Anatomia Patologica, PO Michele e Pietro Ferrero	Verduno (Cuneo), Italy	Biopsy review	
Chantal	Ducret		MD	Urologia, PO Umberto Parini	Aosta, Italy	Provision of patients	
Stefania	Erra		MD	Anatomia Patologica, PO Santo Spirito -	Casale Monferrato (Alessa	Biopsy review	
Ubaldo	Familiari		MD	Anatomia Patologica, PO Umberto Parini	Aosta, Italy	Biopsy review	
Nicola	Faraone		MD	Urologia, PO Humanitas Gradenigo	Torino, Italy	Provision of patients	
Antonella	Ferro		HSD	Urologia, PO Martini	Torino, Italy	Data collection and assembly	
Elda	Feyles		MD	Anatomia Patologica, PO Cardinal Massaia	Asti, Italy	Biopsy review	
Alessandro	Fornari		MD	Anatomia Patologica, AOU San Luigi Gonzaga e Università di	Orbassano, Italy	Biopsy review	
Giuseppe	Forte		MD	Anatomia Patologica, AO Santa Croce e Carle	Cuneo, Italy	Biopsy review	
Flavio	Fraire		MD	Anatomia Patologica, PO Michele e Pietro Ferrero	Verduno (Cuneo), Italy	Biopsy review	
Alessia	Francese		MSc	Infrastruttura Ricerca Formazione e Innovazione - AO SS Ant	Alessandria, Italy	Data collection and assembly	
Pietro	Gabriele		MD	Radioterapia, Istituto di Candiolo - Fondazione del Piemonte	Candiolo (Torino), Italy	Steering committee	
Andrea	Galla		MD	Radioterapia, Istituto di Candiolo - Fondazione del Piemonte	Candiolo (Torino), Italy	Provision of patients	
Diletta	Garrou		MD	Urologia, PO Rivoli	Rivoli (Torino), Italy	Provision of patients	
Marco	Gatti		MD	Radioterapia, Istituto di Candiolo - Fondazione del Piemonte	Candiolo (Torino), Italy	Provision of patients	
Alessandro	Giacobbe		MD	Urologia, AO Ordine Mauriziano	Torino, Italy	Provision of patients	
Giuseppe	Giacomelli		MD	Urologia, PO Maria Vittoria	Torino, Italy	Provision of patients	
Andrea	Giordano		MD	Urologia, AOU Città della Salute e della Scienza e Università d	Torino, Italy	Provision of patients	
Susanna	Grande		MD	Urologia, PO Rivoli	Rivoli (Torino), Italy	Provision of patients	
Alessia	Guarneri		MD	Radioterapia, Istituto di Candiolo - Fondazione del Piemonte	Candiolo (Torino), Italy	Provision of patients	
Sandro	Guglielmetti		MD	Urologia, Ospedali Riuniti ASL TO4	Ivrea (Torino), Italy	Provision of patients	
Pamela Francesca	Guglielmini		MD	Oncologia, AO SS Antonio e Biagio e Cesare Arrigo	Alessandria, Italy	Data collection	
Maria	Iandolo		MD	Anatomia Patologica, ASL TO3	Pinerolo (Torino), Italy	Biopsy review	
Giuseppe Carlo	Iorio		MD	Radioterapia, AOU Città della Salute e della Scienza e Univers	Torino, Italy	Provision of patients	
Paola	Ivaldi		**MSc**	Epidemiologia dei tumori, AOU Città della Salute e della Scie	Torino, Italy	Communication	
Marisa	Kurti		MD	Urologia, PO Humanitas Gradenigo	Torino, Italy	Provision of patients	
Maria Rosa	La Porta		MD	Radioterapia, Ospedali Riuniti ASL TO4	Ivrea (Torino), Italy	Provision of patients	
Giuliana	Leucci		MD	Urologia, PO Humanitas Gradenigo	Torino, Italy	Provision of patients	
Fabiola	Liberale		MD	Urologia, PO Nuovo Ospedale degli Infermi	Ponderano (Biella), Italy	Provision of patients	
Marco	Lucci Chiarissi		MD	Urologia, PO Michele e Pietro Ferrero	Verduno (Cuneo), Italy	Provision of patients	
Monica	Manassero		HSD	Urologia, PO Michele e Pietro Ferrero	Verduno (Cuneo), Italy	Data collection and assembly	

Supplemental Online Content: Nonauthor Collaborators

*First name, last name, and suffix (if applicable) are required and will appear in PubMed.

*First Name and Middle Initial(s)	*Last Name	*Suffix (eg, Jr, III)	Academic Degrees	Institution	Location (city, state/province, country)	Role or Contribution, eg, chair, principal investigator	Group (if more than 1 Group listed in the byline) and/or Subgroup (eg, Steering Committee)
Claudia	Manini		MD	Anatomia Patologica, PO San Giovanni Bosco	Torino, Italy	Biopsy review	
Marco	Manzo		MD	Urologia, PO Humanitas Gradenigo	Torino, Italy	Provision of patients	
Giansilvio	Marchioro		MD	Urologia, AOU Maggiore della Carità e Università del Piemon	Novara, Italy	Provision of patients	
Mauro	Mari		MD	Urologia, PO Rivoli	Rivoli (Torino), Italy	Provision of patients	
Gloria	Maso		MD	Urologia, PO San Biagio	Domodossola (VCO), Italy	Provision of patients	
Federica	Massa		MD	Anatomia Patologica, AOU San Luigi Gonzaga e Università di	Orbassano (Torino), Italy	Biopsy review	
Massimo	Massarelli		MD	Urologia, Ospedali Riuniti ASL TO4	Ivrea (Torino), Italy	Provision of patients	
Guglielmo	Melloni		MD	Urologia, PO Michele e Pietro Ferrero	Verduno (Cuneo), Italy	Provision of patients	
Marinella	Mistrangelo		MD	AOU Città della Salute e della Scienza di Torino	Torino, Italy	Steering committee	
Chiara	Monagheddu		MD	Medico di medicina generale, ASL Torino 3	Torino, Italy	Study design, e-CRF	
Francesco	Morabito		MD	Urologia, PO Cardinal Massaia	Asti, Italy	Provision of patients	
Gregorio	Moro		MD	Radioterapia, PO Nuovo Ospedale degli Infermi	Ponderano (Biella), Italy	Provision of patients	
Ivano	Morra		MD	Urologia, AO Santa Croce e Carle	Cuneo, Italy	Provision of patients	
Carlo	Negro		MD	Urologia, PO Cardinal Massaia	Asti, Italy	Provision of patients	
Valerio	Olivieri		MD	Urologia, Ospedali Riuniti ASL TO4	Ivrea (Torino), Italy	Provision of patients	
Renzo	Orlassino		MD	Anatomia Patologica, Ospedali Riuniti ASL TO4	Ivrea, Ciriè e Chivasso (Tor	Biopsy review	
Cinzia	Ortega		MD	Oncologia, PO Michele e Pietro Ferrero	Verduno (Cuneo), Italy	Steering committee	
Donatella	Pacchioni		MD	Anatomia Patologica 2, AOU Città della Salute e della Scienza	Torino, Italy	Biopsy review	
Alberto	Pagani		MD	Anatomia Patologica, ASL TO3	Pinerolo (Torino), Italy	Biopsy review	
Marco	Pagano		MD	Anatomia Patologica, PO Michele e Pietro Ferrero	Verduno (Cuneo), Italy	Biopsy review	
Eva	Pagano		MSc	Epidemiologia Clinica e Valutativa, AOU Città della Salute e d	Torino, Italy	Data analysis, Health economist	
Mauro	Papotti		MD	Anatomia Patologica 1, AOU Città della Salute e della Scienza	Torino, Italy	Biopsy review	
Massimo	Pasquale		MD	Urologia, PO Maria Vittoria	Torino, Italy	Steering committee	
Roberta	Patetta		MD	Anatomia Patologica, PO Umberto Parini	Aosta, Italy	Biopsy review	
Valter	Pezzuto		MD	Anatomia Patologica, Ospedali Riuniti ASL TO4	Ivrea, Ciriè e Chivasso (Tor	Biopsy review	
Donato	Randone		MD	Urologia, PO Humanitas Gradenigo	Torino, Italy	Steering committee	
Umberto	Ricardi		MD	Radioterapia, AOU Città della Salute e della Scienza e Univers	Torino, Italy	Steering committee	
Daniele	Ricci		MD	Anatomia Patologica, PO Cardinal Massaia	Asti, Italy	Biopsy review	
Rosalba	Rosato		MSc, PhD	Epidemiologia Clinica e Valutativa, AOU Città della Salute e d	Torino, Italy	Data analysis	
Riccardo	Rossi		MD	Urologia, PO SS Annunziata	Savigliano (Cuneo), Italy	Provision of patients	
Cristina	Rossi		MD	Urologia, PO San Giacomo	Novi Ligure (Alessandria), I	Provision of patients	
Diego	Rosso		MD	Urologia, PO SS Annunziata	Savigliano (Cuneo), Italy	Provision of patients	
Fabio	Saccona		MSc	Epidemiologia Clinica e Valutativa, AOU Città della Salute e d	Torino, Italy	Web site manager, e-CRF production	
Omid	Sedigh		MD	Urologia, PO Humanitas Gradenigo	Torino, Italy	Provision of patients	

Supplemental Online Content: Nonauthor Collaborators
*First name, last name, and suffix (if applicable) are required and will appear in PubMed.

***First Name and Middle Initial(s)**	***Last Name**	***Suffix (eg, Jr, III)**	Academic Degrees	Institution	Location (city, state/province, country)	Role or Contribution, eg, chair, principal investigator	Group (if more than 1 Group listed in the byline) and/or Subgroup (eg, Steering Committee)
Ezio	Talarico		MD	Urologia, PO Umberto Parini	Aosta, Italy	Provision of patients	
Stefano	Taraglio		MD	Anatomia Patologica, PO Martini	Torino, Italy	Biopsy review	
Matteo	Taurino		MD	Urologia, AOU Maggiore della Carità e Università del Piemon	Novara, Italy	Provision of patients	
Salvatore	Treffiletti		MD	Urologia, Ospedali Riuniti ASL TO4	Chivasso (Torino), Italy	Provision of patients	
Marcello	Tucci		MD	Oncologia, PO Cardinal Massaia	Asti, Italy	Steering committee	
Marco	Volante		MD, PhD	Dipartimento di Oncologia, Anatomia Patologica, Università	Orbassano, Italy	Biopsy review	
Carla Angela	Zavattero		MD	Anatomia Patologica, PO SS Annunziata, ASL CN1	Savigliano (Cuneo), Italy	Biopsy review	
Luisa	Zegna		MD	Urologia, PO Nuovo Ospedale degli Infermi	Ponderano (Biella), Italy	Provision of patients	
