# Supplementary material for: Patient and Context Factors in the Adoption of Active Surveillance for Low-Risk Prostate Cancer
Source: JAMA Netw Open. 2023 Oct 17;6(10):e2338039. doi: 10.1001/jamanetworkopen.2023.38039 (PMC10582795; doi:10.1001/jamanetworkopen.2023.38039)
Supplement: Supplement 3. — Data Sharing Statement [file jamanetwopen-e2338039-s003.pdf]

Data Sharing Statement

Ciccone. Patient and Context Factors in the Adoption of Active Surveillance for Low-Risk Prostate Cancer. *JAMA Netw Open*. Published October 17, 2023.
doi:10.1001/jamanetworkopen.2023.38039

Data
Data available: Yes
Data types: Deidentified participant data
How to access data: Request for data must be sent to the corresponding author (giovannino.ciccone@gmail.com)
When available: With publication

Supporting Documents
Document types: Statistical/analytic code
How to access documents: Request for statistical/analytic code must be sent to the corresponding author (giovannino.ciccone@gmail.com)
When available: With publication

Additional Information
Who can access the data: Data will be made available to researchers whose proposed use of the data has been approved
Types of analyses: Data will be made available for individual patient data meta-analysis or for other agreed purposes
Mechanisms of data availability: Data will be made available for approved proposals after the START collaborative group approves data access.
